# Cost-efficiency in Medicaid long-term support services: the role of home and community based services

**DOI:** 10.1186/2193-1801-2-305

**Published:** 2013-07-06

**Authors:** Arpita Chattopadhyay, Yang Fan, Sudip Chattopadhyay

**Affiliations:** Division of General Internal Medicine, Department of Medicine, University of California, San Francisco, CA 94143 USA; Department of Economics, University of Washington, 305 Savery Hall, Seattle, WA 98195 USA; Department of Economics, San Francisco State University, 1900 Holloway Ave., San Francisco, CA 94132 USA

**Keywords:** Medicaid, Long-term care, Long-term support services, Cost efficiency, State variation, Home and community based services

## Abstract

Growth in home and community based services (HCBS) has been implicated in rising long-term care expenditure in the Medicaid program. Its efficiency impact has not been tested. Using Stochastic Frontier Analysis (SFA) and panel data methods, we evaluated the cost efficiency of long-term support services (LTSS) provided by state Medicaid agencies and examined its association with intensity of HCBS use. We compared the efficiency of state funded HCBS programs with federal waiver programs. We found substantial variation in cost efficiency of LTSS programs by states, but all showed improvement over time related to increased HCBS use. Higher participation in federal waivers programs yielded additional improvements in cost-efficiency. Results indicate that increasing HCBS services targeted at “high need” population and developmentally disabled individuals would improve efficiency in LTSS delivery. These results reveal the importance of measuring and comparing efficiencies across Medicaid funded LTSS programs, as we introduce reforms in the LTSS delivery system. We recommend that Medicaid agencies invest in the development of improved data sources for the estimation of cost efficiencies of their programs.

## Introduction

Long-term Support Service (LTSS) represents a disproportionate and growing share of Medicaid expenditure. Over 48% of total Medicaid cost covers 6% of Medicaid beneficiaries receiving LTSS (Kaiser Family Foundation [2011c]). In 2006, Medicaid paid for 40% of the total $178 billion in long-term care expenditure (Kaiser Family Foundation [2011a]). Despite being a low cost alternative to institutional care, recent studies have shown that Medicaid spending on home and community based services (i.e. LTSS provided at home and the community) has grown substantially as a proportion of total Medicaid spending on LTSS, rising at a more rapid rate than expenditure on institutional care. Between 1995 and 2005 contribution of home and community based services (HCBS) in Medicaid spending doubled from 19% of Medicaid LTSS expenditure and 6% of total Medicaid spending to 37% of LTSS expenditure and 12% of total Medicaid spending (Kaye et al. [2009]). The Patient Protection and Affordable Care Act (PPACA), through its State Balancing Incentive Program further strengthen the move toward HCBS for Medicaid eligible elderly and disabled persons. As the health care reform is implemented we will see further expansion of these services.

Despite its popularity, much of the escalation in Medicaid LTSS expenditure has been blamed on the growth of HCBS, primarily through the “wood-work” effect (i.e. by increasing the take-up by large numbers of people who would not be institutionalized, but may be willing to use the more desirable home and community based services, resulting in higher overall LTSS expenditure). Two recent studies (Kaye et al. [2009]; Harrington et al. [2011]) suggest that wood-work effect notwithstanding, HCBS have a dampening effect on Medicaid cost escalation. These findings suggest that HCBS may be associated with overall improvement in efficiency of LTSS delivery by reducing non-productive expenses and waste such as, duplication of services and excess capacity.

We evaluate the above hypothesis by utilizing state-level variation and temporal variation in the relative sizes of HCBS program and cost-efficiency (CE) of state LTSS programs. CE that focuses on the cost side of LTSS delivery (instead of the production side when it is called technical efficiency in the econometric literature) is an important, but underutilized measure of efficiency in LTSS delivery. It is a measure of the proportion of input resources (measured in monetary cost) to the output (beneficiaries served), and determines if the same number of beneficiaries could be served less expensively (McGlynn [2008]). The efficiency metric (CE) is superior to the disaggregated costs used by Harrington et al. ([2011]) in that, instead of picking empirical data points on costs for a single year, our approach allows for a comparison of efficiency across states based on a rigorous econometric model that takes advantage of seven years of empirical data points. Because the efficiency measure we use is relative to the lowest possible cost, it facilitates comparison across states and over time. The Harrington et al. ([2011]) paper which calculates cost per individual in each category of LTSS does not allow for a true comparison between states.

When states are compared using CE, efficient states serve more beneficiaries with the same expenditure. The method has been used previously to study cost efficiency in primary care practices, hospitals and nursing homes, (Gruca and Nath [2001]; Hao and Pegels [1994]; Zuckermann et al. [1994]; Rosko and Chilingerian [1999]; Vitaliano and Toren [2005]; Vitaliano and Toren [1994]), as well as health care systems of nation states (Greene [2004]; Oglobin [2011]; Hollingsworth and Wildman [2003]) and regions within a country (Kathuria and Sankar [2005]). However, the method has not been utilized to analyze the effectiveness of the state provided LTSS. An important objective of this study is to show that it is possible to measure and compare the efficiencies of Medicaid funded LTSS programs across states and over time. This is particularly important now, as we implement changes in LTSS delivery systems under PPACA.

This paper describes the time trend in CE for each state during 1999–2007, a period of rapid growth in HCBS under Medicaid. Next, we evaluate the role of the HCBS as an alternative to the institutional LTSS in improving the delivery of LTSS. Finally, we examine if HCBS funded through the 1915(c) federal waiver plans (waivers) are more cost-efficient than state funded programs.

## Background

Medicaid’s LTSS assist adults with disabilities to conduct their day to day activities. The services range from help with eating, bathing and toileting to assistance with grocery shopping, transportation, medication management. These services can be provided at home and the community (HCBS) or in institutions such as nursing homes and in facilities for people with developmental disabilities (ICFMR). Funding for these services comes jointly from the state and federal governments. The federal government’s contribution is determined by the federal matching assistance percentage that accounts for state variation in income. Although the funding for the programs comes jointly from the federal and state governments, the state Medicaid agency is the decision making unit for all Medicaid provided services in the state, including LTSS and can therefore, be seen as having organized systems that produce LTSS for its beneficiaries.

Responding to patient preferences (Eckert et al. [2004]), court ruling^a^, and cost pressures, states have steadily expanded HCBS as an alternative to expensive, but less desirable, institutional care. Between 1999 and 2007, the size of Medicaid HCBS increased in all 50 states and the District of Columbia. The level and pace of the growth has however, varied. This resulted in substantial heterogeneity among the states in the proportion of Medicaid beneficiaries being served in HCBS within the overall Medicaid LTSS programs (Reinhard et al. [2011]). For example, in 2006 HCBS participation ranged from 87% of total LTSS participation in Alaska to 30% in Indiana. The expenditure on HCBS also varied from state to state. This ranged from 68% of total LTSS cost in Oregon to 12% in Mississippi (Ng et al. [2010]).

States use two principal methods of promoting HCBS. The first is through the 1915 (c) waiver of federal requirements for LTSS eligibility. Theses waivers allow states to provide wide-ranging HCBS to individuals who require institutionalization, and provide substantial flexibility to control costs through the establishment of quotas, restrictive eligibility, and demonstration of cost-neutrality compared to institutionalized individuals (Harrington et al. [2011]). All states except Arizona and Vermont have one or more HCBS waivers in operation. The second mechanism for providing HCBS to Medicaid beneficiaries is through state-only plans which typically provide personal care services and home health services for all persons who meet the state disability and income criteria (Kitchener et al. [2007]).

In addition to directly increasing access to HCBS, states regulate the supply of institutional services through the *Certificate of Need* (CON) program that limits constructions of nursing facilities to demonstrable state needs. This further encourages the use of HCBS compared to institutional services. Thirty seven states currently have CON laws in effect (National Conference of State Legislatures [2012]). Another cost containment strategy that may increase the use of HCBS is to use managed care or capitated payment. Managed care organizations have an incentive to avoid the higher cost of institutional services. Seven states had introduced some form of managed LTSS by 2005 (National Consortium for Health System Development [2006]). The *Program of All*-*Inclusive Care for the Elderly* (PACE) is an example of a fully capitated managed care delivery system meant for Medicare-Medicaid dually eligible persons over age 55. It currently operates in 29 states (National PACE Association [2012]).

## Methods

The state level cost efficiencies are estimated using the stochastic frontier function (SFF) method. The SFF method assumes that a decision making unit, combines inputs (that cost a certain amount) to produce the services. Because we are concerned with cost efficiencies of LTSS provided by state Medicaid agencies, we may assume that the Medicaid agency for each state is the decision making unit for that state’s LTSS program, and has an organized system for providing those services for its beneficiaries. The SFF method follows that of Greene and Segal ([2004]) which also estimates cost efficiency using cross-section-time-series data. However, our method takes further advantage of the panel structure of the state level LTSS data and adopts the random effect models that incorporate time varying nature of the inefficiency (Greene [2005]). The estimation was carried out in two steps. We used the SFF method to estimate the cost efficiencies of states’ in LTSS delivery in the first step. In the second step, we regressed the logarithm of these scores obtained from the first step on the penetration of HCBS, and HCBS waivers following the approach developed by Greene ([2004]). The use of the second step addresses cross-state heterogeneity and effectively overcomes the concerns raised in early research using the SFF analysis in the health care sector (Skinner [1994]; Dor [1994]; Newhouse [1994]). More recently, McGlynn ([2008]) again advocated the SFF analysis in the health care sector. The detailed description of the model and its estimation are discussed below.

### Model specification and estimation

**Figure 1:**
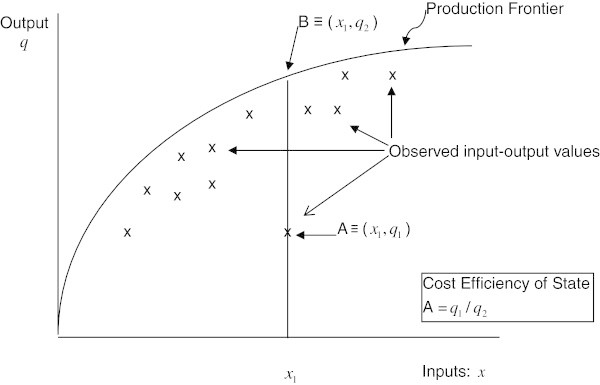
**Cost efficiency in input–output space.**

Cost efficiency (*CE*), in the econometric literature is defined as the ratio of the observed number of beneficiaries served for a given level of inputs and, consequently, for a given cost and the maximum number that could have been served at that cost. Figure 1 illustrates this concept. The solid curve represents the maximum number (*q*) of LTSS clients that can be served by a state for a given level of inputs (*x*). This is the state’s production frontier. The possible input–output combinations in real life however, are always below the frontier and represent varying levels of efficiency.

In Figure 1 the point A (*x*_*1*_,*q*_*1*_) represents the observed output *q*_*1*_ for a given input vector (*x*_*1*_) and the point B (*x*_*1*_,*q*_*2*_) represents the efficient level of output serving a larger number of clients *q*_*2*_ but using the same level of input x_1_. A state’s CE is defined as the ratio *q*_*1*_/*q*_*2*_ ([Fare et al. 1994]).

In general, the production function of LTSS services in a state is represented by the equation:1

where *q* is the number of LTSS clients served by the state Medicaid agency, *x* is the vector of inputs, and *β* is a vector of parameters to be estimated. The function *f*(*x*,*β*) represents the optimal number of LTSS users who can be served with *x* resources, while *CE* representing a state’s cost efficiency is such that 0 ≤ *CE* ≤ 1. Using the theory of duality, the production frontier is transformed to a cost frontier2

here *w* is the vector of input prices and *c*(*w*) is the unit cost function.

### Cost function specification

We assume that each state produces two types of long-term care services, institutional care and non-institutional care (HCBS). Each state also faces a fixed set of input prices, such as wages and costs of facility usage that determine the cost of providing LTSS to the Medicaid enrollees.

Applying the two-output cost function, proposed by Burgess ([1974]) and later used by Truett and Truett ([2003]), the cost function that we estimate for our 50-states’ LTSS production is:3

In the above specification, *q*_*1*_ and *q*_*2*_ represent the numbers of clients in institutional care and HCBS, respectively and *ϵ* = *v* + *u* is the error term consisting of a random component (*v*), and an independently distributed cost efficiency term (*u* = | log(*CE*)|) (Aigner et al. [1977]). This specification is the most appropriate because there are no restrictions on the parameters (Christensen and Greene [1976]). Thus, an efficient cost frontier is given by the following4

According to this specification cost is both increasing in outputs and conforming to usual economies and diseconomies of scale reflecting long run average U-shaped cost curves. States are also assumed to be on their long-run cost curves.

In the above formulation, states that achieve this cost-to-output relationship are deemed to be cost efficient, i.e. *log*(*CE*) = 0. In other words, state Medicaid agencies that operate on this cost-frontier are successful in their attempts to maximize output given the cost, but are still subject to stochastic shocks not in their control (Kumbkakar and Lovell [2000]). State Medicaid agencies that are further away from the cost frontier are less efficient in cost minimization (or service maximization) as compared to those that operate relatively closer to the frontier. For inefficient states the absolute value of log (*CE*) can range from 0 to infinity. Thus, states can be ranked by their cost efficiency score of their Medicaid LTSS programs and this variability can provide an insight into factors that may be associated with a state’s level of efficiency in LTSS delivery.

### Estimation of cost efficiency

In the first step estimation, we use the SFF analysis. There is another method used for cost efficiency estimation, namely the Data Envelopment Analysis (DEA) approach. The DEA approach is often criticized (Dor [1994]) because it ignores the statistical distribution of the cost uncertainties and is based solely on the specific data sample at hand. For these reasons, *we chose not to use the DEA approach*, *using instead the SFF method*. We examined three different distributional assumptions of the efficiency term in SFF, namely truncated normal, half-normal, and exponential. However, the parameter estimations of the SFF were similar across different distributional assumptions. We present the results based on truncated normal efficiency. Using panel data for all 50 states and District of Columbia for 9 years, we allowed efficiency to be time-varying and used the random effects approach to estimate it. Specification of the time-varying efficiency was based on the Battese and Coelli ([1988, 1992]) parameterization. This model is more general and inclusive than the models in which efficiencies are forced to be fixed as time changes. The proposed model, however, does not automatically imply that efficiency will improve. It is quite possible that efficiency may decrease, depending upon parameter estimates. In addition to the variables specified in equation (4), we also added eight year dummies to separate out confounding by year.

### Regression specification for estimating the effect of HCBS on cost efficiency

Following Greene ([2004, 2005]) and Greene and Segal ([2004]), we gauge the independent impact of the state practices on its CE for LTSS delivery in the second step estimation. The use of a two-step approach over the single step approach for cost estimation is still debated in the literature. Unfortunately, a single-step approach, although allows estimation of the cost function, it does not allow an analysis of the effect of important policy tools, such as managed care or the effect of waivers versus state plans that are contributing to the inefficiency in service delivery – an important focus of the present study.

In the second estimation step therefore, we regressed the logarithm of the efficiency scores (*CE*), on a set of state LTSS characteristics controlling for socioeconomics. The LTSS characteristics included the percentage of HCBS participants among LTSS users in a state, percentage of waiver participants among HCBS users in a state, the percentage of ICFMR residents among the institutionalized population in the state, presence of managed LTSS and CON program in the state. State population, income, and unemployment were used to control for differences in state socioeconomic conditions.

### Estimation of the effect of HCBS on cost efficiency

In the first step estimation, the estimated random effects model with year dummies produced year specific estimates of CE scores for each state and year. These scores constituted the dependent variable in our second step analysis. Because we have multiple years of data for each state, we examined fixed effects and random effects models to account for state specific unobserved effects in the second step. However, the Hausman ([1978]) test rejected the random effect specification and we chose the fixed effects model. A major strength of the fixed effects approach is that it captures and eliminates all time-constant heterogeneity among states that remain unobserved in the model, which makes the analysis less likely to be biased. The availability of managed care and the implementation of CON, however varied by year for each state, and these were introduced as time varying covariates in the model.

Our primary variables of interest are the size of the HCBS population in a state and its distribution into waiver plans and state-only plans. Recognizing that state economic environment could influence state expenditure in LTSS and therefore, its efficiency score, we controlled for confounders such as, unemployment rate and per capita income. We included the total population in a state as an indicator of the level of service needs. In addition, we introduced a variable capturing the relative size of ICFMR residents to elderly and disabled nursing home residents as a broad measure of service need and the age distribution among the LTSS users. The developmentally disabled/mentally retarded population represents a unique group of LTSS beneficiaries with somewhat different service needs. We controlled for the presence of CON and managed LTSS in a state to capture state level variation in the policy environment likely to influence the size of HCBS and CE of the state. The availability of managed care, and the implementation of CON, however, varied by year for each state. These were introduced as time varying covariates in the model. We report robust standard errors and associated P-values using STATA 10 (StataCorp [2007]).

### Data sources

Data for this study come from several sources. To estimate CE of states’ LTSS programs, we use data on Medicaid LTSS expenditure and beneficiaries from 49 states and the District of Columbia for years 1999–2007. We excluded Arizona from our analysis because LTSS beneficiaries in Arizona receive services from managed care exclusively. Because it operates on a capitated system, data on expenditure by type of service are not available. Vermont also transitioned to an 1115 global waiver in 2005, and we excluded 2006 & 2007 data for Vermont. We chose our study period to reflect the a time of rapid growth in which HCBS coinciding with a range of policy initiatives directed towards re-balancing of Medicaid LTSS away from institutional care. These are also the most recent years with information on number of participants in non-institutional services.

We obtained expenditure data from the quarterly CMS-64 expense report compiled annually by Thomson Reuters available from http://medicaid.gov/Medicaid-CHIP-Program-Information/By-Topics/Data-and-Systems/MBES/CMS-64-Quarterly-Expense-Report.html. These data cover expenditure in both institutional services, such as nursing homes and intermediate care facilities for people with mental retardation (ICFMR), and non-institutional services provided through waivers and state plans. Participant data for institutional care services were downloaded from the CMS MSIS system (http://www.cms.gov/Research-Statistics-Data-and-Systems/Computer-Data-and-Systems/MedicaidDataSourcesGenInfo/MSIS-Mart-Home.html), and non-institutional services are compiled from Kaiser Family Foundation and University of California at San Francisco’s report of CMS form 372 for each State Medicaid director available at http://www.kff.org/medicaid/upload/7720-04.pdf.

Information on the presence of managed LTSS program in a state was culled from the CMS MSIS system. Data on the presence of PACE, and CON in any given year in a state are obtained from agencies that direct and oversee the respective programs. For example, information on the presence of PACE program was gathered from the website for the National Pace Association at http://www.npaonline.org/website/download.asp?id=1741. This information was combined with information from MSIS to determine if a state operated any managed LTSS program. Data on presence of CON was obtained from the website for the National Conference of State Legislatures available at http://www.ncsl.org/default.aspx?tabid=14373. Information on total state population, state per-capita income, and state unemployment rate were drawn from the Census Bureau. These two variables were used primarily as controls to reflect the general economic condition of the states that can potentially affect the cost efficiency in LTSS delivery. All expenditure data are converted to year 2005 dollar equivalent to account for inflation using the CPI as a measure of inflation. We did not use the health inflation measure because it seemed unlikely that personal care services would experience the high inflation rate of the health care sector, where cost escalation has been attributed primarily to expensive new medical technology (Cutler [2001]).

## Results

Table 1 presents aggregate data in the country over the years 1999–2007 excluding Arizona and Vermont in 2006 and 2007. States spent on average $1.71 billion on long-term care services. The number of participants in institutional setting was lower than the number using HCBS (46% vs. 54%; p < .001) during 1999–2007. Waiver participants represent almost half of all HCBS users during this period. Twenty four of the 50 states had managed LTSS or a *PACE* program in place by 2007; thirty six states had *CON* laws for nursing home beds.

**Table 1:** **Descriptive statistics (N=448)**

Variable	Mean	Std. deviation	Minimum	Maximum
*Total cost (in billion$)*	1.71	2.49	0.1	18.8
*Efficiency score*	34.88	29.05	8.92	198.17
*HCBS participation (%)*	53.90	13.28	20.08	89.50
*HCBS participants in waiver (%)*	52.14	20.23	0	94.00
*ICFMR among Institutionalized (%)*	5.70	4.13	0	16.73
*Managed care (% of states)*	0.42	0.49	0	1.00
*CON (% of states)*	0.74	0.44	0	1.00
*Unemployment rate (%)*	4.70	1.15	2.30	8.10
*Per capita income($)*	33,234	5,495	2,3617	57,455
*Population (in thousands)*	5,713	6,412	480	36,600

Table 2 provides the results from the first step of our analysis, i.e. the estimated cost frontier function.

**Table 2:** **Estimated Stochastic frontier function model**^**a**^**used to derive cost efficiency**

Variables^b^	Coefficient estimate	Standard error
log hcbs	0.22^***^	0.03
Log institutional	0.17^*^	0.04
Yr. 1999	−0.31^***^	0.09
Yr. 2000	−0.25^***^	0.08
Yr. 2001	−0.19^***^	0.06
Yr. 2002	−0.10^**^	0.06
Yr. 2003	−0.10^**^	0.04
Yr. 2004	−0.05	0.04
Yr. 2005	−0.03	0.03
Yr. 2006	−0.02	0.02
constant	13.68^**^	1.50

Note: ^a^ Frontier estimation model is truncated normal efficiency & time varying decay.

^b^ Dependent variable is the logarithm of total cost (log cost).

*** Significant at 1% level; ** Significant at 5% level; * Significant at 10% level.

We ranked the cost efficiency score, – measured as the log of the independently distributed cost efficiency term (*u* = | log(*CE*)|) - estimated from the aforementioned first step of the estimation process, for each of the 50 states for each of the years from best to worst. Cost efficiencies vary considerably across states and across years (see Table 3). Note that higher values indicate inefficient states. We find that smaller states like Wyoming, South Dakota, Idaho, and Arkansas, are among the ten most efficient states; whereas the states like California, New York, and Pennsylvania are in the bottom of the list. However, all states show a gradual decrease in scores, implying a steady increase in cost-efficiency over the years. The national average efficiency scores are also reported in the last row of Table 3. This trend during a period when HCBS has also been steadily growing agrees with other studies (Kaye et al. [2009]; Harrington et al. [2011]) and provides additional evidence of the effectiveness of HCBS as a LTSS cost containment strategy.

**Table 3:** **Efficiency scores**^**a**^**of State long-term care programs 1999-2007**

State	Cost efficiency								
	1999	2000	2001	2002	2003	2004	2005	2006	2007
High efficiency states									
WY	9.6	9.5	9.4	9.3	9.2	9.1	9.1	9.0	8.9
VT	9.9	9.8	9.7	9.6	9.5	9.5	9.4		
SD	10.2	10.1	10.1	10.0	9.9	9.8	9.7	9.6	9.5
NV	10.6	10.5	10.4	10.4	10.3	10.2	10.1	10.0	9.9
MT	12.2	12.1	12.0	11.9	11.8	11.7	11.6	11.5	11.4
ID	12.4	12.2	12.1	12.0	11.9	11.8	11.7	11.6	11.5
UT	13.4	13.3	13.2	13.0	12.9	12.8	12.7	12.5	12.4
DE	13.9	13.7	13.6	13.5	13.3	13.2	13.1	12.9	12.8
HI	13.9	13.8	13.6	13.5	13.4	13.2	13.1	13.0	12.8
AK	14.6	14.5	14.3	14.2	14.0	13.9	13.8	13.6	13.5
ND	14.8	14.6	14.5	14.3	14.2	14.0	13.9	13.8	13.6
DC	14.9	14.7	14.5	14.4	14.3	14.1	14.0	13.8	13.7
Moderate efficiency states									
NH	19.0	18.8	18.5	18.3	18.1	17.9	17.7	17.5	17.4
ME	20.1	19.9	19.6	19.4	19.2	19.0	18.8	18.6	18.4
AR	20.5	20.3	20.1	19.8	19.6	19.4	19.2	19.0	18.7
RI	20.7	20.4	20.2	20.0	19.8	19.5	19.3	19.1	18.9
NM	21.0	20.8	20.5	20.3	20.1	19.8	19.6	19.4	19.2
NE	22.0	21.7	21.4	21.2	20.9	20.7	20.5	20.2	20.0
WV	23.4	23.1	22.9	22.6	22.3	22.1	21.8	21.5	21.3
OK	24.1	23.8	23.5	23.2	22.9	22.6	22.4	22.1	21.8
MS	24.7	24.4	24.1	23.8	23.5	23.2	22.9	22.6	22.4
KS	24.8	24.5	24.2	23.9	23.7	23.4	23.1	22.8	22.5
SC	25.5	25.2	24.9	24.6	24.3	24.0	23.7	23.4	23.1
CO	25.9	25.6	25.3	25.0	24.7	24.4	24.1	23.8	23.5
Low efficiency states									
IA	26.3	25.9	25.6	25.3	25.0	24.7	24.4	24.1	23.8
OR	26.3	26.0	25.7	25.4	25.1	24.8	24.5	24.2	23.9
KY	26.9	26.5	26.2	25.9	25.6	25.3	24.9	24.6	24.3
AL	31.9	31.5	31.1	30.7	30.3	29.9	29.5	29.1	28.7
MO	33.0	32.5	32.1	31.7	31.3	30.8	30.4	30.0	29.7
GA	34.8	34.3	33.9	33.4	33.0	32.5	32.1	31.7	31.3
VA	35.3	34.8	34.3	33.9	33.4	33.0	32.5	32.1	31.7
WA	38.7	38.1	37.6	37.1	36.6	36.1	35.6	35.1	34.6
LA	39.2	38.6	38.1	37.6	37.1	36.5	36.0	35.6	35.1
MD	39.4	38.9	38.3	37.8	37.3	36.8	36.3	35.8	35.3
IN	43.5	42.8	42.2	41.6	41.0	40.5	39.9	39.3	38.8
WI	45.0	44.4	43.7	43.1	42.5	41.9	41.3	40.7	40.1
Very low efficiency states									
TN	45.4	44.7	44.1	43.5	42.8	42.2	41.6	41.0	40.4
MI	47.3	46.6	45.9	45.2	44.6	43.9	43.3	42.7	42.1
IL	48.6	47.8	47.1	46.4	45.8	45.1	44.4	43.8	43.2
NC	48.6	47.9	47.2	46.5	45.8	45.2	44.5	43.9	43.2
CT	49.3	48.6	47.9	47.2	46.5	45.8	45.1	44.5	43.8
MN	52.4	51.6	50.9	50.1	49.3	48.6	47.9	47.2	46.5
TX	53.6	52.8	52.0	51.2	50.5	49.7	49.0	48.2	47.5
FL	55.9	55.0	54.2	53.4	52.5	51.8	51.0	50.2	49.5
MA	59.3	58.4	57.5	56.6	55.7	54.9	54.0	53.2	52.4
NJ	68.9	67.8	66.7	65.6	64.6	63.5	62.5	61.6	60.6
OH	74.8	73.5	72.3	71.2	70.0	68.9	67.8	66.7	65.6
CA	79.8	78.4	77.1	75.9	74.6	73.4	72.2	71.0	69.9
PA	117.6	115.5	113.4	111.3	109.4	107.4	105.5	103.6	101.8
NY	198.2	194.2	190.3	186.5	182.8	179.2	175.7	172.2	168.9
All USA	36.8	36.3	35.8	35.2	34.7	34.2	33.7	33.2	32.8

Note: ^a^ Higher scores correspond to lower efficiency.

**Figure 2:**
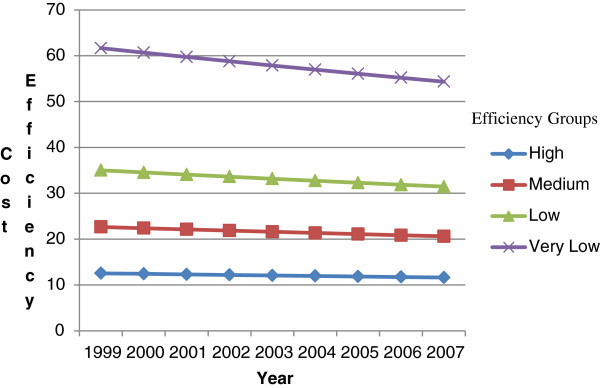
**Increasing cost efficiency in LTSS over time.**

Figure 2 charts the CE scores of states grouped by their efficiency quartiles over the 9-year period. We find that while all states show improvements in cost efficiency over time, the least efficient states improved the most.

Recognizing that many factors may be contributing to the variation in cost-efficiencies among the states, we present below the results of the multivariable regression analysis, obtained in the second step, to assess the importance of HCBS program variables in the determination of a state’s efficiency level.

### State policies and the efficiency in care delivery

The results from the second step regression model are presented in Table 4. A negative and highly significant coefficient for share of HCBS users suggests that an increase in HCBS participation is associated with improvements in efficiency of LTSS delivery. All else being equal, a 1 percentage point increase in the share of HCBS participants in a state results in a decrease in score (implying increase in cost-efficiency) by 30% (1-*e*^-*0*.*37*^ = 0.30; p < .001). HCBS services delivered through federal *waiver* programs promote additional efficiencies. One percentage point rise in the proportion of HCBS participants in *waiver* programs compared to state funded programs would result in an additional 11% (1-*e*^-*0*.*11*^ = .89; p < .001) increase in the cost-efficiency.

**Table 4:** **Coefficient estimates from multivariable regression model of cost efficiency scores**

Variable name^a^	Fixed effects model	95% Confidence interval^b^
*Share of HCBS*	-.37***	-.43, -.31
*% Waiver among HCBS*	-.11***	-.16, -.06
*% in ICFMR among Institutionalized*	.77*	.14 1.40
*Managed Care*	-.01**	-.02 -.003
*CON*	.00	-.02, .01
*ln(population)*	-.00	-.00, .00
*ln(per capita income)*	-.05**	-.08 -.01
*Unemployment rate*	-.01*	-.01 -.00
Constant	4.02***	3.60, 4.44
Within R^2^ =	0.62	

Notes:

^a^ Dependent variable is the log of efficiency scores; higher scores correspond to lower efficiency.

^b^ Confidence Intervals account for within state clustering.

*** Significant at <0.1% level; ** Significant at 1% level; * Significance at 5% level.

*HCBS* Home and community based services.

Waiver: 1915(c) federal waiver.

*CON* Certificate of Need.

Although, HCBS improves efficiency in LTSS generally, this form of delivery would be particularly effective when targeted toward the developmentally disabled/mentally retarded Medicaid beneficiaries residing in ICFMRs as reflected in the coefficient for percentage ICFMR. This coefficient shows that an increase in one percentage in the proportion of ICFMR residents among institutionalized persons is associated with 215% (e^0.77^ = 2.15; p < .05) decline in efficiency (see Table 4).

The presence of managed care in a state is associated with a small, but significant improvement in cost efficiency. Other significant determinants of efficiency in LTSS delivery are income and unemployment rate of a state, justifying the introduction of these two variables as controls to reflect the general economic condition of the states that can potentially affect the cost efficiency in LTSS delivery.

## Discussion

States have been moving toward increased delivery of LTSS through HCBS. In addition, PPACA provides states with additional opportunities to provide access to HCBS services for Medicaid-only beneficiaries and dual eligible with LTSS needs. Policymakers investing funds in these innovations hope that they can reduce the challenges beneficiaries with LTSS needs experience in obtaining the services they need while maximizing their choice of providers and remaining in home and community-based settings. Policymakers also hope that such investments will contain Medicaid and Medicare spending growth in the face of growing pressure on these programs to deliver services to an aging population.

Unlike traditional source of demand, government-sponsored Medicaid LTSS programs represent coverage-by-necessity whereby individuals must demonstrate their need for coverage through income requirements and/or disability. As fiscal pressures rise, there is principally only one way to reduce cost; that is by reducing waste and non-productive expenses.

In this article, we have presented our analysis of interstate variation in cost efficiency (CE) of Medicaid LTSS delivery. Estimates reveal substantial variation in CE of state LTSS systems resulting from differences in the volume and type of services provided by each state. The study shows that even while the total expenditure grew, all 50 states show improved cost-efficiency in LTSS delivery during a period when HCBS had grown by 48% (Kaiser Family Foundation [2011b]). Regression results show that larger proportion of participants in HCBS was associated with greater cost efficiency.

Although, HCBS is in general associated with more efficient LTSS delivery, there is substantial variation in its effect across states. States with large HCBS programs tend to be lower on the efficiency scale. One possible explanation could be that larger programs make the management of services difficult, leading to waste and duplication. Another explanation could be in the funding mechanism that is used by states to provide HCBS. An examination of state efficiency scores and the size of waiver programs indicate that states with higher proportion of HCBS users in waiver programs are more cost-efficient than states with smaller HCBS waiver programs. For example, states in top efficiency levels, namely, Wyoming and Nevada have respectively, 85% and 50% of their HCBS users in waiver programs; whereas, states in the bottom ten in the efficiency ranking, namely California and New York have 15% and 28% of HCBS beneficiaries in waivers, respectively. Results from multivariable regression corroborate this finding.

The second reason why states with large state-funded HCBS programs experience lower efficiency could be because of problems in targeting. Since state programs require that services be provided for all eligible individuals, states are limited in their ability to carve-out services by geography or cost. Waiver programs require that the participants be nursing home certifiable and cost neutral. Waivers, therefore ensure the substitution of high cost institutional service with less expensive HCBS. Correct targeting has long been recognized as an important ingredient for achieving cost effectiveness in HCBS (Grabowski [2006]; Kemper [1988]).

Though managed care represents a fairly small portion of state LTSS programs, the significant and positive coefficient of managed LTSS shows that the presence of capitated LTSS improves the overall efficiency of LTSS delivery in a state. This study further shows that efficiency gains attributable to managed long-term care, spill over an entire state. Many managed care recipients are PACE participants who are dually eligible and probably the frailest group of Medicaid LTSS users. As in the case of HCBS waivers, PACE participants qualify for Medicaid institutional level of care. PACE capitation rates are adjusted upwards for higher levels of frailty of this population compared to other long-term care users. The higher capitation rate, however, is compensated for by improvements in the overall cost-efficiency of Medicaid LTSS programs.

Statistically non-significant effect for the CON on CE is supported by other studies (Miller et al. [2005]; Grabowski et al. [2003]), which have found that states that repealed the CON program, saw little to no change in their cost structure. This coupled with the fact that the estimated cost function showed that increase in stitutional care resulted in declining cost upto a certain point, and increasing after that, which suggests that nursing homes and ICFMR may already be operating at less than full capacity. We examined the effect of CON in states with and without a moratarium on institutional care and found the effect of CON to be similar in both types of states.

Although, the findings in our study are supported by rigorous empirical evidence, there are a few shortcomings. For example, we did not consider cross-state variation in quality of LTSS in calculating the efficiency scores. A concern that is often raised is that increased quality of support services may negate efficiency gains achieved by states, if high quality is associated with high cost resulting in inefficiency (Newhouse [1994]). In other words, there might be a negative correlation between quality and efficiency. If these variations are random across states, the random effects model utilized in the first step regression can fully capture and eliminate them. If these variations are not random, some unobserved factors will remain, and our results may be biased. In order to address this important issue, we further investigated the relationship between quality rankings and efficiency rankings of the states. Since data on quality of care are not available for our study period, we could not include quality in our model to directly estimate the effect of quality on efficiency. Instead we utilized data on quality rankings available for year 2011 reported in Reinhard et al. ([2011]), and the efficiency rankings of states obtained from our study to carry out Spearman’s rank test for any evidence of significant negative ranked correlation. Contrary to the general belief, the statistical test suggested that LTSS quality was, in fact, *positively* associated with cost-efficiency (Spearman rank correlation 0.38; p-value = 0.006). Hence, this result points to evidence that by not adjusting for quality our regression results are, in fact, conservative estimates.

Other limitations are lack of data on intensity of use (length of stay, hours of service etc.), and other LTSS policies adopted by states such as, cost sharing and consumer direction may have confounded our results in evaluating the associations between HCBS and cost efficiencies in the second step regression analysis. To the extent that the values of these omitted variables do not change in a state within our study period, the fixed effects estimation technique used in the second step analysis can fully capture and eliminate them. If these variables have changed over time, the second step regression model would not capture them.

## Conclusion

Despite a few limitations, this paper demonstrates the efficiency impact of HCBS. It advances the field of LTSS delivery systems by revealing which policies may be associated with efficient programs. Our study shows that of the two broad strategies policymakers can adopt to improve access to HCBS, namely provide more funds for zhome and community-based services by expanding Medicaid coverage and state-funded home care programs, and liberalizing waiver programs for nursing home eligible persons, the second strategy is more efficient. Other initiatives to reduce the incremental cost of LTSS, suggested by this study are adoption of managed LTSS and increasing HCBS for developmentally disabled and mentally retarded beneficiaries.

Perhaps most importantly, the study advances the field of LTSS program evaluation by providing a tool to policy makers to monitor the relative efficiency of various LTSS programs. The study shows that it is possible to measure and compare the efficiency of Medicaid provided LTSS across states and over time. This is particularly important as the US implements health system reforms. We intend to extend this analysis with finer grained data on beneficiary characteristics and quality outcomes, as such data become available. We hope that this study encourages state Medicaid agencies to invest in the development of improved data sources and estimation methods, with the overall objective to stimulate action that will improve the performance of LTSS systems and contribute to improving the welfare of people.

## Endnotes

^a^ In *Olmstead v*. *L*.*C*. *and E*.*W*., *1999*, the Supreme Court ruled that the "integration mandate" of the Americans with Disabilities Act requires public agencies to provide services "in the most integrated setting appropriate to the needs of qualified individuals with disabilities.

## Abbreviations

HCBS: Home and community based services
LTSS: Long-term support services
PPACA: Patient protection and affordable care act
CE: Cost-efficiency
ICFMR: Intermediate care facilities for people with mental retardation
DEA: Data envelopment analysis
SFF: Stochastic frontier function.
